# Prenatal Folinic Acid in FRAA-Positive Pregnancies: Preschool Neurodevelopmental Outcomes from a Pilot RCT Follow-Up

**DOI:** 10.3390/nu18182970

**Published:** 2026-09-10

**Authors:** Claudio Giorlandino, Alvaro Mesoraca, Katia Margiotti, Marco Fabiani, Marina Cupellaro, Francesca Giorlandino, Maria Luisa Mastrandrea, Raffaella Raffio, Francesco Pignataro, Lucia Mangiafico, Laura D’Emidio, Claudio Coco, Vincenzo Milite

**Affiliations:** 1 Human Genetics Lab, ALTAMEDICA, Viale Liegi 45, 00198 Rome, Italy; 2 Department of Prenatal Diagnosis, Fetal-Maternal Medical Centre, ALTAMEDICA, Viale Liegi 45, 00198 Rome, Italy

**Keywords:** autism spectrum disorder, folate receptor alpha autoantibodies, folinic acid, prenatal supplementation, preschool neurodevelopment, cerebral folate deficiency

## Abstract

**Background**: Folate receptor alpha autoantibodies (FRAAs) during pregnancy impair placental and cerebral folate transport, predisposing the developing brain to autism spectrum disorder (ASD) through cerebral folate deficiency (CFD). A previously published pilot randomised controlled trial (RCT) showed that prenatal calcium folinate (folinic acid), compared with standard folic acid, lowered ASD incidence in the offspring of FRAA-positive mothers at 24–30 months. Mechanistically, whereas folic acid depends largely on the antibody-blocked folate receptor alpha, folinic acid (a reduced folate) enters cells through the reduced folate carrier and proton-coupled folate transporter, restoring cerebral folate delivery despite receptor blockade. We investigated whether this benefit persists at preschool age (36–50 months); the primary endpoint was verification that children born without ASD remain ASD-free. **Methods**: Pre-planned longitudinal follow-up of the 18 mother–child pairs who completed the original pilot RCT. Sixteen families (88.9%) were re-evaluated at a mean age of 45.4 ± 3.8 months (folinic acid, *n* = 9; folic acid, *n* = 7) using DSM-5-TR diagnosis, ADOS-2, ADI-R, WPPSI-IV, and Vineland-3, alongside standardised language and learning-prerequisite batteries. **Results**: The pre-specified primary endpoint was met: none of the eight folinic acid-group children who were ASD-free at 24–30 months developed ASD (0/8; 95% CI 0–36.9%). With only eight children in this subgroup, the estimate is inevitably imprecise, and we read it as pilot evidence of stability rather than proof of complete protection. Confirmed ASD at preschool age was 1/9 (11.1%) versus 5/7 (71.4%; *p* = 0.024). Folinic acid-exposed children showed lower ADOS-2 scores (*p* = 0.008), higher WPPSI-IV Full Scale IQ (*p* < 0.001), and superior Vineland-3 Adaptive Behaviour Composite scores (*p* = 0.002). Trajectory analysis showed neurodevelopmental stability in the folinic acid group versus progressive worsening in the folic acid group (*p* = 0.043). **Conclusions**: Children born without ASD to FRAA-positive mothers prenatally supplemented with folinic acid remained ASD-free at preschool age; the single folinic acid-group ASD case was attributable to a pathogenic SHANK2 variant. These findings support prenatal folinic acid as a targeted neuroprotective strategy in FRAA-positive pregnancies and warrant confirmation in larger, multicentre randomised trials.

## 1. Introduction

Autism spectrum disorder (ASD) is a prevalent and clinically heterogeneous neurodevelopmental condition characterised by persistent deficits in social communication and interaction, alongside restricted, repetitive patterns of behaviour and interests [1]. Its global prevalence now approximates 1 in 36 children in some populations [2] and continues to rise, placing an increasing burden on affected individuals, families, and healthcare systems. ASD is recognised as a multifactorial disorder in which genetic, immune, metabolic, and environmental factors converge during critical windows of prenatal and early postnatal brain development.

Among the immune-mediated pathogenic mechanisms implicated in ASD, folate receptor alpha autoantibodies (FRAAs) have received increasing scientific attention [3,4,5,6]. These immunoglobulins, predominantly of the IgG class, target the folate receptor alpha (FRα), which is highly expressed in the placenta, choroid plexus, and brain microvasculature. Blocking FRAAs prevents folate from binding FRα, thereby impairing transcytosis of 5-methyltetrahydrofolate (5-MTHF) across the placental and blood–brain barriers. Binding FRAAs may additionally induce receptor internalisation or complement-mediated inflammation, further compromising cerebral folate delivery [7]. The net result is cerebral folate deficiency (CFD), characterised by low cerebrospinal fluid 5-MTHF concentrations despite normal systemic folate status [7]. CFD has been associated with a spectrum of neurological manifestations including ASD, infantile-onset CFD syndromes, spastic-ataxic syndromes, and Rett syndrome [6,7].

Epidemiological studies have identified FRAA positivity in 60–75% of mothers of children with ASD [8,9,10], as well as in a substantial proportion of affected children themselves, suggesting both prenatal immunoglobulin transfer and postnatal endogenous autoantibody production as pathogenic mechanisms. In the preconception population, FRAA positivity has been identified in approximately 17% of women screened in a randomised controlled trial (RCT) [11].

Folinic acid (leucovorin; calcium folinate), an active reduced-form folate that bypasses FRα-mediated transport by utilising the reduced folate carrier (RFC) and the proton-coupled folate transporter (PCFT) [5,10], represents a pharmacologically rational strategy to restore adequate cerebral folate availability in the presence of FRAA-mediated receptor blockade.

We recently published the first randomised controlled trial (RCT) evaluating prenatal folinic acid versus folic acid supplementation in FRAA-positive pregnancies [11]. In that single-centre, double blind pilot study (ISRCTN96269039), 18 mother–child pairs completed the trial, and at 24–30 months prenatal folinic acid was associated with a significantly lower ASD incidence together with milder ADOS-2 severity and higher cognitive scores than folic acid. However, the observation window of the original pilot study was limited to 24–30 months, an age at which ASD diagnosis, language profile, adaptive functioning, and cognitive trajectories are still evolving and variably stable [12,13].

The present study reports the preschool follow-up of the same original randomised pilot cohort, testing two co-primary endpoints: (i) whether children born without ASD to FRAA-positive mothers prenatally supplemented with folinic acid remain free from ASD at preschool age; and (ii) whether children born FRAA-positive but without ASD, when treated postnatally with folinic acid, maintain normal neurodevelopmental trajectories. Secondary endpoints address the full spectrum of cognitive, adaptive, language, and learning outcomes at preschool age, as well as longitudinal developmental trajectories. This follow-up does not simply repeat the original comparison. Its purpose is to find out whether the early protective signal holds at an age when the diagnosis of ASD is much more stable. A diagnosis made at 24–30 months is provisional: some children thought to be unaffected in the second year later meet criteria, and a few early diagnoses remit. We therefore asked whether the children born free of ASD after prenatal folinic acid were still free of it at preschool age, and whether their cognitive, adaptive and language development had stayed on track, expecting meanwhile that the folic acid group would show emerging or worsening difficulties. Confirming that no neurodevelopmental disorder had appeared at this later and more reliable stage seemed to us essential before an early benefit could be called lasting.

## 2. Materials and Methods

### 2.1. Study Design

This is a longitudinal follow-up of a previously published single-centre, double-blind pilot RCT (ISRCTN96269039) [11]. The present report constitutes a prospective observational cohort study conducted on the 18 mother–child pairs who completed the original trial. Ethics Committee approval was obtained as an amendment to the original approval. All participating families provided updated written informed consent prior to preschool assessment. At the preschool assessments, the psychologists who administered and scored the tests had not taken part in the original randomisation and did not know the prenatal treatment allocation, which had stayed concealed since the double-blind parent trial. We cannot claim that masking was complete: the child’s clinical history was available to the team, and a test such as the ADOS-2 necessarily exposes the child’s behaviour during administration, so blinding to a previous ASD diagnosis could not be guaranteed for the diagnostic measures. For the cognitive, adaptive and language scales, the use of standard norm-referenced scoring left little room for observer bias. This follow-up was therefore not a blinded or double-blind study; it is best regarded as an open, observational assessment in which only the original treatment allocation remained concealed.

### 2.2. Study Population and Follow-Up Retention

The follow-up cohort comprised all 18 mother–child pairs who completed the original pilot RCT: 10 in which the mother received prenatal calcium folinate 15 mg/day and 8 in which the mother received prenatal folic acid 400 μg/day, both administered orally once daily from the first trimester to delivery. All 18 families were contacted for preschool re-evaluation; families unable to attend their scheduled appointment were offered alternative slots. Sixteen of 18 families (88.9%) were successfully re-evaluated, yielding a final preschool cohort of 9 children in the prenatal folinic acid group and 7 in the prenatal folic acid group. Two families were lost to follow-up: one from the folinic acid group (family relocated to a different region) and one from the folic acid group (family declined further participation for personal reasons unrelated to the intervention).

Both children lost to follow-up had been classified as ASD-free at the original 24–30 months assessment. Their withdrawal therefore did not alter the proportion of children with ASD available for preschool assessment in either group, and loss to follow-up was non-differential with respect to the primary outcome, making directional bias unlikely. Consequently, the 9 children available for preschool follow-up in the folinic acid group comprised the single child diagnosed with ASD at 24–30 months and 8 children who had been classified as ASD-free, making the primary endpoint verification of neurodevelopmental stability in the ASD-free subgroup. The 7 children in the folic acid group comprised 5 with confirmed ASD and 2 without ASD at the original assessment.

**Figure 1:**
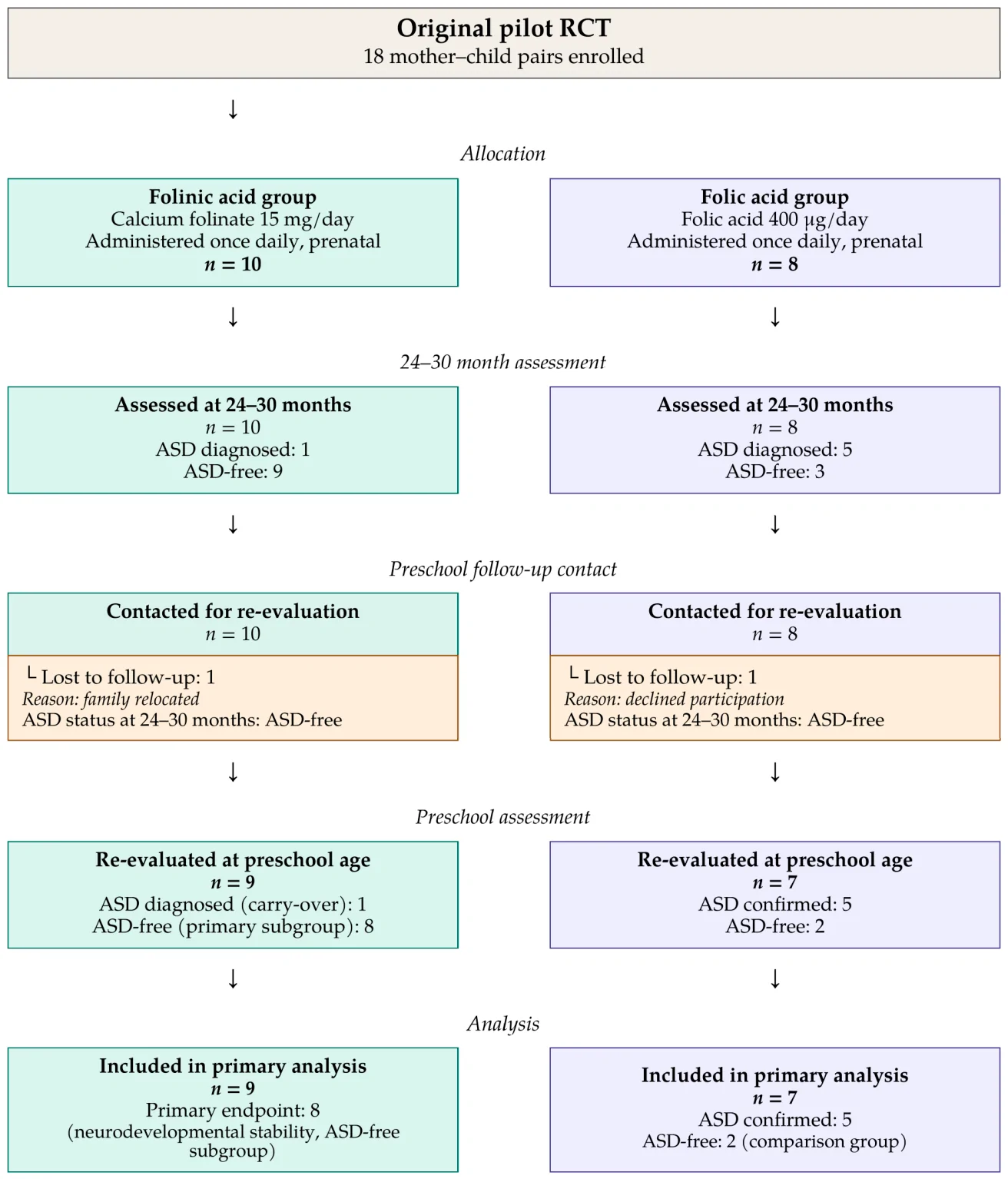
Participant flow diagram. Note: Both children lost to follow-up were classified as ASD-free at the original 24–30 month assessment. Loss to follow-up was non-differential with respect to the primary outcome; directional bias is therefore unlikely. ASD = autism spectrum disorder; ^L^ LTFU = lost to follow-up. Overall retention rate: 16/18 (88.9%).

A participant flow diagram is presented in Figure 1.

### 2.3. Follow-Up Assessment Window

Preschool follow-up assessments were conducted between January 2025 and March 2026, when children were aged 36–50 months (preschool epoch). The mean age at assessment was 45.6 ± 3.9 months in the folinic acid group and 45.1 ± 3.7 months in the folic acid group (*p* = 0.79), indicating comparable assessment timing. The mean inter-assessment interval from the original 24–30 month evaluation to preschool follow-up was 18.2 ± 3.0 months. This age window was selected because ASD diagnosis achieves its highest stability after 36 months [13,14] and cognitive and language profiling attain substantially greater reliability and clinical informativeness than at the toddler epoch. The primary endpoint of this follow-up study was the proportion of children in the prenatal folinic acid group who had been classified as ASD-free at 24–30 months and remained free from ASD at preschool follow-up. The co-primary endpoint was the neurodevelopmental outcome at preschool follow-up in the two children of the folinic acid group who had tested FRAA-positive in the neonatal period (without an ASD diagnosis) and were subsequently started on postnatal oral calcium folinate at 1 mg/kg/day, a dose consistent with published paediatric therapeutic guidelines for CFD [15].

### 2.4. Secondary Outcomes

Secondary outcomes included: (i) confirmed ASD diagnosis at preschool age according to DSM-5-TR criteria, classified as persistent, remitted, newly diagnosed, or absent; (ii) ASD severity assessed by ADOS-2 and ADI-R; (iii) cognitive ability assessed by WPPSI-IV or, for younger or lower-functioning children, Bayley-4 or Mullen Scales; (iv) adaptive behaviour assessed by Vineland-3; (v) standardised language evaluation (PVB, TCGB batteries); (vi) preschool learning prerequisites (TPV and PRCR-2 batteries); and (vii) within-subject change in ADOS-2 total score and cognitive composite score from the original 24–30 month assessment to preschool age.

Assessment instruments. All measures were standardised tests, administered and scored by trained clinical psychologists according to their manuals, using the validated Italian version and norms where these exist. ASD diagnosis and severity were based on the ADOS-2 (Italian edition) [16] and the ADI-R [17]. Cognitive ability was assessed with the WPPSI-IV (ages 2 years 6 months to 7 years 7 months, in its Italian standardisation) [18] and, for children whose age or developmental level made it unsuitable, with the Bayley-4 [19] or the Mullen Scales [20]. Adaptive functioning was measured with the Vineland-3 [21], language with the PVB and TCGB, and preschool learning prerequisites with the TPV and PRCR-2. The primary validation and Italian normative references for each test are listed in the References.

### 2.5. FRAA Testing at Follow-Up

Maternal and child FRAA levels were re-measured at preschool follow-up in available serum samples using the same validated ELISA-based assay (ELK Biotechnology Co., Ltd., Wuhan, Hubei, China; positivity threshold ≥ 30 ng/mL) as in the original study [11]. Maternal FRAA titres were obtained from 13 mothers (7 folinic acid group, 6 folic acid group); child FRAA status at follow-up was assessed in 11 children (6 folinic acid group, 5 folic acid group), subject to parental consent. Longitudinal changes in maternal titres from preconception to preschool follow-up were described.

### 2.6. Postnatal Folinic Acid Supplementation

Postnatal folinic acid supplementation was not prescribed as part of the original randomised intervention or the current follow-up protocol. It was initiated on a purely clinical basis, independent of the research programme. In the folinic acid group, 2 of 9 children received postnatal calcium folinate (leucovorin) at a dose of 1 mg/kg/day orally, initiated after confirmation of neonatal FRAA positivity without an ASD diagnosis and continued through the preschool follow-up period. In the folic acid group, 4 of 7 children received postnatal folinic acid (median dose 5 mg/day; median start age 18 months; median duration 16 months), predominantly in the context of ASD diagnosis with clinical suspicion of CFD. Postnatal folinic acid use was recorded as an exploratory clinical exposure variable, and its potential contribution to preschool outcomes was evaluated in pre-specified sensitivity analyses.

### 2.7. Statistical Analysis

Categorical variables were compared using Fisher’s exact test. Continuous variables were tested for normality using the Shapiro–Wilk test; normally distributed variables were compared with the independent-samples Student’s *t*-test and non-normally distributed variables with the Mann–Whitney U test. Within-subject changes from 24 to 30 months to preschool age were evaluated using the Wilcoxon signed-rank test. Effect sizes are reported as Cohen’s d for continuous outcomes and odds ratios (OR) with 95% confidence intervals (CI) for binary outcomes. A two-tailed *p* < 0.05 was considered statistically significant. Given the exploratory nature of this follow-up and the small sample size, *p*-values are reported without correction for multiple comparisons. Accordingly, all secondary and subgroup analyses are regarded as exploratory and hypothesis-generating, and their *p*-values should be read as descriptive rather than confirmatory. All analyses were performed using IBM SPSS Statistics (version 27.0; IBM Corporation, Armonk, NY, USA) and Python (version 3.10; Python Software Foundation, Wilmington, DE, USA), using the SciPy and pandas libraries. Two pre-specified sensitivity analyses were conducted: (1) primary ASD outcome analysis restricted to children who had not received postnatal folinic acid supplementation for ≥4 consecutive weeks, to assess the independence of preschool outcomes from postnatal treatment; (2) exploratory logistic regression for ASD diagnosis at preschool age, including postnatal folinic acid supplementation as a binary covariate. Given the severely reduced sample size in these analyses, results are considered hypothesis-generating and are intended to inform the design of future adequately powered trials rather than to support independent inferential conclusions. No formal sample-size calculation was carried out. The cohort was fixed by the parent trial and comprised all the children who completed it, so the study was meant to estimate effects and generate hypotheses rather than to test them; we express precision through effect sizes and confidence intervals, which we give for every estimate, including the mean differences and Cohen’s d and not only the odds ratios. Change within each child between the two assessments was tested with the Wilcoxon signed-rank test. We did not fit linear mixed models or generalised estimating equations: with only two time points, a random-intercept model comes down to essentially the same change analysis, and with sixteen children the variance estimates of these methods, and the GEE sandwich estimator in particular, would be unreliable. The logistic regression is reported only to show that the direction of effect agrees with the other analyses; with one event in the folinic acid group and five in the folic acid group it lies close to separation, and its estimate is unstable, so the categorical inference rests on Fisher’s exact test.

## 3. Results

### 3.1. Follow-Up Retention and Cohort Composition

Of the 18 children who completed the original pilot RCT, 16 (88.9%) underwent complete preschool assessment: 9 in the prenatal folinic acid group and 7 in the prenatal folic acid group. The two children lost to follow-up (one per group) were both ASD-free at the original assessment; their loss is therefore unlikely to have biased the primary outcome comparison in either direction (Figure 1). Mean ages at preschool assessment were 45.6 ± 3.9 months (folinic acid) and 45.1 ± 3.7 months (folic acid) (*p* = 0.79), confirming balanced assessment timing. The mean inter-assessment interval was 18.2 ± 3.0 months.

Regarding sex composition, the folinic acid preschool group (*n* = 9) comprised 5 males, of whom one had ASD, and 4 females. The folic acid preschool group (*n* = 7) comprised 4 males and 3 females; ASD was diagnosed in 3 of the males and 2 of the females (5 cases in total). The single ASD case in the folinic acid group was therefore a male, whereas in the folic acid group ASD affected both sexes.

### 3.2. Neurodevelopmental Stability in the ASD-Free Folinic Acid Group

This is the central finding of the present study. All 8 children in the prenatal folinic acid group who had been classified as ASD-free at the 24–30 months assessment remained free from ASD at preschool follow-up (0/8; 95% CI 0–36.9%). The pre-specified endpoint was therefore met. With only eight children, the interval is wide, so we regard this as pilot evidence of stability rather than proof of complete protection. This finding directly answers the primary clinical question: children born without ASD to FRAA-positive mothers prenatally supplemented with folinic acid do not develop ASD at preschool age. No ASD diagnoses emerged de novo in either group.

Of equally high clinical relevance is the outcome of the two children in the folinic acid group who had tested FRAA-positive in the neonatal period (presumably reflecting maternally transferred IgG via a microchimerism mechanism) but had not received an ASD diagnosis. Both children, initiated on postnatal oral calcium folinate at 1 mg/kg/day, maintained normal neurocognitive development across all assessed domains at preschool follow-up, meeting no diagnostic criteria for ASD, language disorder, or clinically significant developmental delay. This co-primary endpoint was therefore also met.

One child in the folinic acid group showed early, subclinical signs of learning difficulties at preschool assessment (difficulties in short-term verbal memory and phonological awareness), not meeting full DSM-5-TR criteria for a specific learning disorder, but warranting longitudinal monitoring into school age.

### 3.3. ASD Diagnostic Status at Preschool Age: Full Cohort Comparison

At preschool follow-up, the proportion of children with a confirmed ASD diagnosis according to DSM-5-TR criteria was 1/9 (11.1%) in the prenatal folinic acid group and 5/7 (71.4%) in the prenatal folic acid group (Fisher’s exact test, *p* = 0.024; OR = 0.056, 95% CI: 0.004–0.732). These results are presented in Table 1. The odds ratio comes from an exploratory logistic model; with only six events in all, it lies close to separation and is therefore imprecise, and we rely on Fisher’s exact test for the categorical comparison.

The single ASD case in the folinic acid group is the same male child identified at 24–30 months, in whom the original pilot study had documented a pathogenic variant in the SHANK2 gene [11]. His ASD presentation remained clinically stable at preschool follow-up, and his case is most plausibly attributable to a genetic mechanism independent of the folate pathway, as discussed in the original report. In the folic acid group, all five children originally diagnosed with ASD at 24–30 months retained a confirmed ASD diagnosis at preschool follow-up. Among these, four showed stability or worsening of the clinical profile, while one child demonstrated partial symptomatic improvement (lower ADOS-2 score relative to the original assessment) but continued to meet full DSM-5-TR diagnostic criteria.

Learning disorders at preschool age were present in 1/9 (11.1%) children in the folinic acid group and 5/7 (71.4%) in the folic acid group (Fisher’s exact test, *p* = 0.024; OR = 0.056, 95% CI: 0.004–0.732). One child in the folic acid group received a learning disorder classification at preschool age who had not met criteria at the original 24–30-month assessment (Table 1).

### 3.4. ASD Severity: ADOS-2 and ADI-R at Preschool Age

ADOS-2 total scores at preschool follow-up were significantly lower in the prenatal folinic acid group (median [IQR] = 3.5 [2–5]) compared with the prenatal folic acid group (10.5 [9–12]) (Mann–Whitney U test, *p* = 0.008; Cohen’s d = −1.72), indicating a large, statistically robust between-group difference (Table 2). ADI-R domain scores across Social Interaction, Communication, and Restricted/Repetitive Behaviour subscales were consistently and significantly lower in the folinic acid group.

### 3.5. Cognitive Outcomes: WPPSI-IV

WPPSI-IV Full Scale IQ scores at preschool follow-up were significantly higher in the prenatal folinic acid group (mean ± SD = 106.4 ± 6.1) than in the prenatal folic acid group (90.2 ± 8.8) (Student’s *t*-test, *p* < 0.001; Cohen’s d = 2.18). The magnitude of this difference exceeds that observed at 24–30 months (Cohen’s d = 1.53; Bayley-4 Cognitive Composite), suggesting progressive cognitive divergence between groups rather than attenuation of the early advantage. Significant between-group differences were present across all WPPSI-IV index scores, including Verbal Comprehension, Visual Spatial, Fluid Reasoning, Working Memory, and Processing Speed indices, indicating pervasive rather than domain-specific cognitive advantage in the folinic acid group (Table 2). The groups differed by 16.2 Full Scale IQ points (95% CI 7.5 to 24.9; Cohen’s d = 2.18, 95% CI 0.95 to 3.44).

### 3.6. Adaptive Behaviour: Vineland-3

Vineland-3 Adaptive Behaviour Composite (ABC) scores were significantly higher in the folinic acid group (mean ± SD = 103.2 ± 6.8) compared with the folic acid group (85.4 ± 9.6) (Student’s *t*-test, *p* = 0.002; Cohen’s d = 2.11). Significant differences were observed across the Communication (*p* = 0.004), Daily Living Skills (*p* = 0.008), and Socialisation (*p* = 0.010) domains. The Motor Skills domain did not reach statistical significance (*p* = 0.068), consistent with the expected relative preservation of motor development in ASD (Table 2). The Adaptive Behaviour Composite differed by 17.8 points (95% CI 8.3 to 27.3; Cohen’s d = 2.11, 95% CI 0.87–3.35).

### 3.7. Language Profile and Preschool Learning Prerequisites

Standardised language assessment revealed significant between-group differences in expressive language (standard score: 99.3 ± 8.1 vs. 83.7 ± 11.4; *p* = 0.007; Cohen’s d = 1.63) and receptive language (101.8 ± 7.3 vs. 86.1 ± 10.7; *p* = 0.005; Cohen’s d = 1.73) favoring the folinic acid group. Pre-literacy and pre-numeracy composite scores were likewise significantly higher in the folinic acid group. These findings suggest that the cognitive and language benefits associated with prenatal folinic acid exposure extend to measurable improvements in academic readiness at preschool age (Table 2). Expressive language differed by 15.6 points (95% CI 4.3 to 26.9; d = 1.62, 95% CI 0.48 to 2.75) and receptive language by 15.7 points (95% CI 5.2 to 26.2; d = 1.76, 95% CI 0.60 to 2.92). The effect sizes are large but, as the intervals show, estimated with little precision.

### 3.8. Longitudinal Developmental Trajectory Analysis

Within-subject trajectory analysis from the original 24–30 month assessment to preschool follow-up demonstrated neurodevelopmental stability in the folinic acid group and progressive divergence in the folic acid group. ADOS-2 total scores in the folinic acid group showed a median change of −1.0 (range: −3 to +1; Wilcoxon signed-rank test, *p* = 0.18), consistent with a stable, non-deteriorating neurodevelopmental course. In contrast, the folic acid group demonstrated a statistically significant worsening of ADOS-2 scores (median change +1.5, range: −1 to +4; Wilcoxon *p* = 0.043), reflecting progressive autistic symptom severity over the inter-assessment interval. The full trajectory dataset is presented in Table 3 and Figure 1.

Cognitive composite scores showed a mean increase of +2.2 points over the inter-assessment period in the folinic acid group (8/9 children showed stable or improved scores), whereas the folic acid group showed a mean decline of −5.2 points (5/7 children showed a decrease). This progressive divergence in both behavioural and cognitive trajectories suggests that the folic acid group is not merely delayed relative to the folinic acid group but is following a qualitatively distinct developmental course, consistent with the natural history of inadequately corrected CFD-related neurodevelopmental impairment.

### 3.9. FRAA Status at Preschool Follow-Up

Maternal FRAA titres at preschool follow-up were significantly lower in the folinic acid group (mean ± SD = 66.4 ± 21.3 ng/mL) compared with the folic acid group (89.1 ± 18.7 ng/mL; *p* = 0.038), despite all tested mothers remaining FRAA-positive (titre ≥30 ng/mL) in both groups. The clinical significance of this titre difference is uncertain and requires prospective evaluation with systematic immunological monitoring in larger cohorts.

Among the 11 children tested at follow-up, FRAA positivity was identified in 2/6 (33.3%) in the folinic acid group and 4/5 (80.0%) in the folic acid group (Fisher’s exact test, *p* = 0.24). The non-significant trend is likely attributable to insufficient statistical power given the small number tested. Notably, the persistence of positive FRAA titres in children at more than 4 years of age may indicate endogenous autoantibody production rather than merely the residual persistence of maternally transferred IgG, a crucial finding that necessitates future longitudinal research incorporating paired IgG subclass analysis.

### 3.10. Postnatal Folinic Acid Supplementation

At the time of preschool follow-up, 6 of 16 children had received postnatal folinic acid: 2/9 (22.2%) in the folinic acid group and 4/7 (57.1%) in the folic acid group (Fisher’s exact test, *p* = 0.16). In the folinic acid group, both postnatally supplemented children had confirmed FRAA positivity without an ASD diagnosis and received oral calcium folinate at 1 mg/kg/day; both maintained normal neurodevelopment at preschool follow-up.

In the folic acid group, four postnatally supplemented children received folinic acid at a median dose of 5 mg/day primarily in the context of ASD and clinical CFD suspicion. Intriguingly, only one of these four children demonstrated mild, partial clinical improvements—specifically a modest reduction in the ADOS-2 Social Affect subdomain and subtle gains in expressive language skills—whereas the remaining three children exhibited stable or worsening symptomatology. Crucially, all four children ultimately retained their full confirmed diagnostic classification of ASD at the preschool assessment. Full postnatal supplementation data are presented in Table 4.

In the pre-specified sensitivity analysis excluding postnatally supplemented children (folinic acid group: *n* = 7; folic acid group: *n* = 3), the proportion with confirmed ASD was 1/7 (14.3%) in the folinic acid group versus 1/3 (33.3%) in the folic acid group (Fisher’s exact test, *p* = 0.47). Although this analysis is severely underpowered due to the sample reduction, the directional consistency with the primary analysis indicates that the preschool outcomes primarily reflect prenatal intervention rather than postnatal supplementation. These sensitivity findings should be interpreted exclusively as hypothesis-generating for future trial design.

## 4. Discussion

The present study constitutes the first preschool follow-up of a randomised pilot trial of prenatal folinic acid versus folic acid supplementation in FRAA-positive pregnancies [11]. Its principal and most clinically salient finding is the confirmation of the primary endpoint: all 8 children in the prenatal folinic acid group who had been classified as ASD-free at 24–30 months remained free from ASD at preschool age (0/8; 0%). This suggests that the early protective signal associated with prenatal folinic acid supplementation in FRAA-positive pregnancies is not a transient developmental advantage but represents durable neurodevelopmental stability extending to a clinically more robust and diagnostically stable developmental epoch.

The biological plausibility of this sustained benefit is well grounded in the neurobiological consequences of folate availability during gestation. Folate, in its bioactive form 5-MTHF, is an obligate cofactor for DNA synthesis, epigenetic methylation of CpG sites and histones, and one-carbon metabolic pathways that sustain neurogenesis, axonal myelination, and cortical organisation during the second and third trimesters of pregnancy. FRAA-mediated blockade of FRα during gestation impairs 5-MTHF delivery to the fetal central nervous system, potentially programming durable epigenetic alterations in neural circuits subserving social cognition, language, and executive function. Conversely, restoration of adequate 5-MTHF delivery through folinic acid—which bypasses FRα blockade by utilising RFC- and PCFT-mediated transport [10,11] during the prenatal critical window may support more resilient neural architecture and more favourable neurodevelopmental trajectories that manifest progressively as the child’s phenotype unfolds through the preschool years.

At the cellular level, the mechanistic basis for this protective effect lies in the capacity of folinic acid to restore intracellular one-carbon flux within the developing fetal brain despite FRα blockade. Whereas both blocking and binding FRAA subtypes interfere with the high-affinity transport of 5-MTHF across the choroid plexus epithelium into the cerebrospinal fluid, disrupting cerebral folate delivery during critical windows of fetal neurodevelopment, folinic acid (5-formyltetrahydrofolate) enters the central nervous system through alternative, FRα-independent routes principally the proton-coupled folate transporter (PCFT/SLC46A1) and the reduced folate carrier (RFC/SLC19A1), both expressed at the choroid plexus and blood–brain barrier and characterised by low affinity for FRAAs. This allows adequate intracellular folate delivery to be maintained even in the presence of receptor-blocking autoantibodies [10,11].

The trajectory analysis points to a durable rather than transient effect. The folinic acid group demonstrated stable ADOS-2 scores over the inter-assessment interval (median change −1.0; Wilcoxon *p* = 0.18), whereas the folic acid group showed a statistically significant progressive worsening (median change +1.5; Wilcoxon *p* = 0.043). This divergence in trajectories is consistent with the natural history of inadequately corrected CFD, in which progressive cerebral folate insufficiency during synaptic pruning and myelination phases leads to expanding rather than static neurodevelopmental impairment [6,7]. The parallel progressive decline in cognitive composite scores in the folic acid group (−5.2 points mean) versus the gain in the folinic acid group (+2.2 points) reinforces this interpretation.

The single ASD case observed in the folinic acid group merits separate consideration. This male child, the sole ASD case in the group at both the original and preschool assessments, was found in the original pilot study to carry a pathogenic variant in the SHANK2 gene [11]. As previously discussed, this case is most plausibly attributable to a folate-pathway-independent genetic aetiology, given the complex and multifactorial nature of ASD. Its persistence is therefore not informative regarding the efficacy of the prenatal intervention and reinforces, rather than weakens, the interpretation that the between-group differences reflect the immuno-nutritional mechanism targeted by folinic acid. Given that this is a single case within a subgroup of only nine children, its influence on group proportions is inevitably large, and it should therefore be interpreted with caution. The case is also worth describing in its own right. SHANK2 codes for a scaffolding protein that holds the machinery of excitatory synapses in place, and a pathogenic variant produces a synaptic disorder that has nothing to do with folate metabolism or with folate-receptor antibodies. SHANK2 (also known as ProSAP1) maps to chromosome 11q13 and encodes a core postsynaptic-density scaffolding protein of glutamatergic excitatory synapses; loss-of-function variants disrupt dendritic-spine maturation and glutamatergic signalling and are an established monogenic cause of autism spectrum disorder, frequently accompanied by intellectual disability and language impairment [22]. Such a cell-autonomous synaptic mechanism plausibly accounts for the early onset and the stability of this child’s ASD across the 24–30-month and preschool assessments, independently of prenatal folate status. This child’s autism is, in effect, genetically determined and belongs to a different mechanism from the folate-responsive cases, which is why his outcome tells us nothing about whether the treatment works and, if anything, sharpens the contrast with the other children. It is a reminder that in these pregnancies metabolic and immune testing should go together with a proper genetic work-up (chromosomal microarray and exome sequencing where indicated), so that a monogenic cause is recognised for what it is rather than mistaken for a treatment that has failed.

The co-primary endpoint of sustained normal neurodevelopment in the two FRAA-positive children without ASD who received postnatal folinic acid at 1 mg/kg/day is clinically relevant for two reasons. First, it suggests that neonatal FRAA positivity, likely reflecting maternally transferred IgG rather than endogenous production at this age, does not inevitably progress to ASD when folate receptor-bypassing supplementation is initiated promptly. Second, it provides a rationale for systematic postnatal FRAA screening and early folinic acid supplementation in neonates born to FRAA-positive mothers, a strategy analogous to the post-partum immunological monitoring already established in other maternal IgG transfer syndromes. The paediatric dose of 1 mg/kg/day used in these two children is at the lower end of the evidence-based therapeutic range (0.5–2.0 mg/kg/day) and was well tolerated, consistent with the established safety profile of leucovorin in paediatric populations.

The sensitivity analysis excluding postnatally supplemented children, while severely underpowered (folinic acid: *n* = 7; folic acid: *n* = 3), yielded directionally consistent results (ASD: 1/7 vs. 1/3), suggesting that the preschool benefit is not principally attributable to postnatal folinic acid use and predominantly reflects the prenatal intervention. This inference is further supported by the observation that among the postnatally supplemented children in the folic acid group, only one child demonstrated mild clinical improvements (specifically a modest reduction in the ADOS-2 Social Affect subdomain and small gains in expressive language), while all four children ultimately retained their confirmed ASD diagnoses. This clinical pattern is highly consistent with the existing literature [5], which underscores that while postnatal folinic acid can alleviate specific core symptoms of ASD by optimising remaining metabolic pathways, it cannot fully compensate for or reverse the foundational neurodevelopmental trajectory established during the irreplaceable prenatal critical window. Postnatal supplementation at this dose and duration was insufficient to reverse early neurodevelopmental divergence, reinforcing the critical-window hypothesis for the primary protective effect.

We were not able to record the behavioural, speech and educational support the children received after birth, and for the continuous outcomes this is a real limitation. Because such services are provided according to need, they were probably more intensive in the folic acid group, where most children had ASD, than in the folinic acid group, where only one did; if anything, this would have narrowed the gap we observed rather than created it. The primary endpoint is largely shielded from the problem, since it concerns children who were already free of ASD and so fell outside the pathways that trigger intensive intervention, and the children in the folic acid group who did receive postnatal folinic acid kept their diagnoses, which shows that later treatment did not undo an established course. We therefore see the prenatal intervention as the main, though not the only, explanation for the difference in diagnosis, while accepting that the size of the differences on the continuous measures cannot be fully separated from differences in postnatal care.

The emergence of one additional learning disorder diagnosis in the folic acid group at preschool age, not identified at the original 24–30-month assessment, is consistent with the clinical hypothesis that subthreshold manifestations of CFD-associated neurodevelopmental impairment become detectable only as preschool academic demands increase sensitivity to deficits in working memory, phonological processing, and language. This finding underscores the importance of extending follow-up into school age, when learning disorders achieve their full diagnostic expression and impact.

The significant reduction in maternal FRAA titres in the folinic acid group at preschool follow-up (66.4 vs. 89.1 ng/mL; *p* = 0.038) is an exploratory but hypothesis-generating observation. Because folate is the entry point of one-carbon metabolism and sustains the synthesis of S-adenosyl-methionine (SAM), the universal methyl donor, adequate folate availability raises the intracellular SAM/S-adenosyl-homocysteine (SAM/SAH) ratio, the cellular “methylation index” that sets the capacity for DNA and histone methylation [23]. A plausible epigenetic route is therefore that sustained folate sufficiency, by supporting this methylation capacity, modulates the methylation status of the FOLR1 promoter, which encodes folate receptor alpha (FRα), the very antigen targeted by FRAAs. Increased FOLR1 promoter methylation could down-regulate FRα expression and thereby reduce the amount of antigen available to drive autoantibody production, offering a mechanism by which folate status could attenuate FRAA titres over time [24,25]. We emphasise that this is a mechanistic hypothesis: causal directionality cannot be established in this observational follow-up, confounding by unmeasured immunological factors cannot be excluded, and whether maternal FRAA titres are shaped by, or merely reflect, folate-dependent methylation would require epigenome-wide methylation profiling of paired maternal and child samples. Because we measured FRAA titres only, without immune-cell subtyping or regulatory T-cell profiling, we do not claim that folinic acid directly regulates autoantibody production; the lower titres in the folinic acid group are reported as an association whose mechanism remains to be established.

## 5. Limitations

The limitations of this preschool follow-up must be explicitly acknowledged. First and foremost, the sample size is small (*n* = 16 at follow-up), substantially limiting statistical power, precluding multivariate adjustment, and increasing the probability of both type I and type II errors; all findings must be interpreted as exploratory and hypothesis-generating. Accordingly, all effect estimates are imprecise, with wide confidence intervals and a non-negligible risk of chance findings, and every between-group difference should be interpreted with corresponding caution. Second, the per-protocol analytical approach, inherited from the original pilot study, may overestimate treatment effects relative to an intention-to-treat analysis. Third, the single-centre design and socioeconomic and cultural context of Rome, Italy (including regional differences in genetic background, baseline folate status and screening practice), limit generalisability to other populations, healthcare systems, and FRAA screening programmes. Fourth, the FRAA assay used is a research-use-only ELISA not yet harmonised across multiple laboratories; the positivity threshold of 30 ng/mL, while internally validated, requires standardisation for multicentre use. Fifth, assessor blinding at preschool follow-up could not be guaranteed in all cases, introducing a potential risk of observer bias, particularly in the open clinical assessment of language and learning prerequisites. This risk is most relevant to the observer-rated ADOS-2 and could, in principle, influence the between-group comparison of ADOS-2 scores. Sixth, postnatal developmental interventions—including early behavioural therapies, speech and language therapy, and educational support—were not systematically recorded and may have differentially benefited children in the folic acid group. Seventh, the absence of neuroimaging data (MRI, DTI) and electrophysiological measures (EEG, ERP) limits mechanistic interpretation of the group differences. Eighth, the short total follow-up duration does not permit evaluation of school-age outcomes, including academic performance, executive function, or social adaptation. Ninth, the exploratory logistic regression is based on very few outcome events and lies close to separation; it is therefore unstable and prone to overfitting, and the categorical inference relies on Fisher’s exact test rather than on the model estimates. These limitations underscore the imperative for adequately powered, multicentre, blinded, randomised trials with controlled postnatal exposure and extended longitudinal observation into school age.

## 6. Conclusions

This preschool follow-up of the first randomised pilot trial of prenatal folinic acid in FRAA-positive pregnancies suggests that the early neurodevelopmental benefit seen at 24–30 months may be durable and extends into a clinically more stable and diagnostically more informative developmental epoch. The pre-specified endpoint was met in every case examined (0/8 conversions; 95% CI 0–36.9%): all 8 children born without ASD to FRAA-positive mothers prenatally supplemented with folinic acid remained free from ASD at preschool age. Developmental trajectory analysis indicates progressive divergence between groups, with stability in the folinic acid group and worsening ASD severity in the folic acid group, a pattern mechanistically consistent with inadequately corrected prenatal CFD.

The single ASD case in the folinic acid group is attributable to an independent genetic cause (a pathogenic SHANK2 variant documented in the original pilot study), supporting the interpretation that the between-group differences are attributable to the immuno-nutritional intervention rather than to differential heritable risk. Children born FRAA-positive but without ASD who received postnatal calcium folinate at 1 mg/kg/day maintained normal neurodevelopment at preschool follow-up, providing preliminary evidence for the clinical utility of postnatal FRAA screening and targeted folinic acid supplementation in this immunologically defined subgroup.

If confirmed in larger cohorts, these findings would support prenatal folinic acid supplementation as a targeted neuroprotective strategy in FRAA-positive pregnancies, analogous in concept to periconceptional folic acid supplementation for neural tube defect prevention and would provide a rationale for incorporating routine FRAA screening into preconception care programmes. We stress, however, that universal preconception FRAA screening cannot be justified by this pilot follow-up alone and would require validation in adequately powered, multicentre randomised trials before any clinical implementation. Definitive conclusions await confirmation in larger, multicentre, prospective randomised trials with adequate statistical power, blinded follow-up assessors, controlled postnatal exposure, and extended longitudinal observation into school age, including evaluation of dose–response relationships, optimal supplementation duration, and the predictive value of FRAA titre and antibody subtype. Because the sample is small and the design exploratory, these results are best treated as hypothesis-generating. The exploratory analyses, and the logistic regression in particular, rest on very few events and are unstable, so we make no firm causal or mechanistic claim at this stage.

## Figures and Tables

**Table 1:** Longitudinal ASD and learning disorder outcomes at 24–30 months (original assessment) and preschool follow-up.

Outcome	Folinic Acid 24–30 mo (*n* = 10)	Folic Acid 24–30 mo (*n* = 8)	Folinic Acid Preschool (*n* = 9)	Folic Acid Preschool (*n* = 7)
ASD diagnosis confirmed, *n* (%)	1 (10.0%)	5 (62.5%)	1 (11.1%)	5 (71.4%)
Fisher’s exact test (*p*); OR (95% CI)	*p* = 0.043; OR = 0.067 (0.005–0.823)		*p* = 0.024; OR = 0.056 (0.004–0.732)	
Learning disorder, *n* (%)	1 (10.0%)	4 (50.0%)	1 (11.1%)	5 (71.4%)
ADOS-2 total score, median [IQR]	4.5 (3–5)	9 (8–10)	3.5 (2–5)	10.5 (9–12)

Categorical outcomes were compared using Fisher’s exact test; ADOS-2 total scores were compared using the Mann–Whitney U test. The odds ratio refers to the odds of ASD in the prenatal folinic acid group relative to the folic acid group. No de novo ASD diagnosis emerged at preschool follow-up in either group. ASD: autism spectrum disorder; ADOS-2: Autism Diagnostic Observation Schedule, Second Edition; IQR: interquartile range; OR: odds ratio; CI: confidence interval.

**Table 2:** Secondary neurodevelopmental outcomes at preschool follow-up (36–50 months).

Outcome Measure	Folinic Acid (*n* = 9)	Folic Acid (*n* = 7)	*p*-Value	Cohen’s d
**ASD severity**				
ADOS-2 total score, median [IQR]	3.5 [2–5]	10.5 [9–12]	0.008	−1.72
**Cognitive ability (WPPSI-IV)**				
Full Scale IQ, mean ± SD	106.4 ± 6.1	90.2 ± 8.8	<0.001	2.18
**Adaptive behaviour (Vineland-3)**				
Adaptive Behaviour Composite, mean ± SD	103.2 ± 6.8	85.4 ± 9.6	0.002	2.11
Communication domain	—	—	0.004	—
Daily Living Skills domain	—	—	0.008	—
Socialisation domain	—	—	0.010	—
Motor Skills domain	—	—	0.068	—
**Language profile (standard scores)**				
Expressive language, mean ± SD	99.3 ± 8.1	83.7 ± 11.4	0.007	1.63
Receptive language, mean ± SD	101.8 ± 7.3	86.1 ± 10.7	0.005	1.73

Data are presented as median [interquartile range] for non-normally distributed variables and mean ± standard deviation for normally distributed variables. Between-group comparisons were performed using the Mann–Whitney U test (ADOS-2) and the independent-samples Student’s t-test (WPPSI-IV, Vineland-3, language scores). Effect sizes are expressed as Cohen’s d; a negative value for ADOS-2 indicates lower symptom severity in the folinic acid group. WPPSI-IV, Vineland-3, and language scores are standardised to a population mean of 100 (SD 15). ASD: autism spectrum disorder; ADOS-2: Autism Diagnostic Observation Schedule, Second Edition; IQR: interquartile range; SD: standard deviation; WPPSI-IV: Wechsler Preschool and Primary Scale of Intelligence, Fourth Edition.

**Table 3:** Longitudinal developmental trajectory: within-group changes from 24–30 months to preschool follow-up.

Parameter	Folinic Acid 24–30 mo (*n* = 9)	Folinic Acid Preschool (*n* = 9)	Folic Acid 24–30 mo (*n* = 7)	Folic Acid Preschool (*n* = 7)
ADOS-2 total, median [IQR]	4.5 [3–5]	3.5 [2–5]	9 [8–10]	10.5 [9–12]
Within-group change (Wilcoxon *p*)	—	*p* = 0.18	—	*p* = 0.043
Cognitive composite, mean (SD)	104.2 (±5.8)	106.4 (±6.1)	95.4 (±6.1)	90.2 (±8.8)
Mean change	—	+2.2	—	−5.2

IQR: interquartile range; SD: standard deviation. Within-group changes were evaluated using the Wilcoxon signed-rank test (ADOS-2 total score) and the paired-samples *t*-test (cognitive composite). Cognitive composite refers to the Bayley-4 Cognitive Composite at 24–30 months and the WPPSI-IV Full Scale IQ at preschool follow-up. Both indices are standardised to a population mean of 100 (SD 15) and are therefore directly comparable across time points.

**Table 4:** Postnatal folinic acid supplementation data (exploratory clinical exposure variable) °.

Variable	Folinic Acid Group (*n* = 2)	Folic Acid Group (*n* = 4)
Dose, median [range]	1 mg/kg/day (both children)	5 mg/day [2–10]
Start age (months), median [range]	18 [14–22]	18 [12–24]
Duration (months), median [range]	15 [10–24]	16 [8–28]
Indication: ASD + CFD suspicion, *n* =	0	4
Indication: FRAA positivity without ASD, *n* =	2	0

ASD: autism spectrum disorder; CFD: cerebral folate deficiency; FRAA: folate receptor alpha autoantibody. ° Postnatal folinic acid was not a protocol-specified intervention; data reflect real-world clinical prescribing decisions made independently of the research programme. The two FRAA-positive children without ASD in the folinic acid group received calcium folinate at 1 mg/kg/day.

## Data Availability

The original contributions presented in this study are included in the article. Further inquiries can be directed to the corresponding author.

## Abbreviations

5-MTHF, 5-methyltetrahydrofolate; ABC, Adaptive Behaviour Composite; ADI-R, Autism Diagnostic Interview–Revised; ADOS-2, Autism Diagnostic Observation Schedule, Second Edition; ASD, autism spectrum disorder; CFD, cerebral folate deficiency; CI, confidence interval; DSM-5-TR, Diagnostic and Statistical Manual of Mental Disorders, Fifth Edition, Text Revision; ELISA, enzyme-linked immunosorbent assay; FRAA, folate receptor alpha autoantibody; FRα, folate receptor alpha; IgG, immunoglobulin G; IQR, interquartile range; OR, odds ratio; PCFT, proton-coupled folate transporter; PRCR-2 and TPV, standardised Italian preschool learning-prerequisite batteries; PVB and TCGB, standardised Italian language batteries; RCT, randomised controlled trial; RFC, reduced folate carrier; SD, standard deviation; Vineland-3, Vineland Adaptive Behavior Scales, Third Edition; WPPSI-IV, Wechsler Preschool and Primary Scale of Intelligence, Fourth Edition.
